# Influence of the Composition and Testing Modes on the Electrochemical Performance of Li-Rich Cathode Materials

**DOI:** 10.3390/nano12224054

**Published:** 2022-11-17

**Authors:** Lidia Pechen, Elena Makhonina, Anna Medvedeva, Yury Politov, Aleksander Rumyantsev, Yury Koshtyal, Alexander Goloveshkin, Igor Eremenko

**Affiliations:** 1 Kurnakov Institute of General and Inorganic Chemistry of the Russian Academy of Sciences, 31 Leninsky pr., 119991 Moscow, Russia; 2 Ioffe Institute of the Russian Academy of Sciences, 26 Politekhnicheskaya ul., 194021 St. Petersburg, Russia; 3 A.N. Nesmeyanov Institute of Organoelement Compounds of the Russian Academy of Sciences, 28 Vavilova ul., 119334 Moscow, Russia

**Keywords:** Li-rich cathode material, lithium-ion battery, voltage and capacity fade, testing mode

## Abstract

Li-rich oxides are promising cathode materials for Li-ion batteries. In this work, a number of different compositions of Li-rich materials and various electrochemical testing modes were investigated. The structure, chemical composition, and morphology of the materials synthesized were studied by XRD with Rietveld refinement, ICP-OES, and SEM. The particle size distributions were determined by a laser analyzer. The galvanostatic intermittent titration technique and galvanostatic cycling with different potential limits at various current densities were used to study the materials. The electrochemical study showed that gradual increase in the upper voltage limit (formation cycles) was needed to improve further cycling of the cathode materials under study. A comparison of the data obtained in different voltage ranges showed that a lower cut-off potential of 2.5 V (2.5–4.7 V range) was required for a good cyclability with a high discharge capacity. An increase in the low cut-off potential to 3.0 V (3.0–4.8 V voltage range) did not improve the electrochemical performance of the oxides and, on the contrary, considerably decreased the discharge capacity and increased the capacity fade. The LMR35 cathode material (Li_1.149_Ni_0.184_Mn_0.482_Co_0.184_O_2_) demonstrated the best functional properties among all the compositions studied.

## 1. Introduction

Today, there is continued interest in the development of energy storage devices, especially supercapacitors and lithium-ion batteries (LIBs) [1,2,3]. Li-rich cathode materials for LIBs of the general formula Li_1+x_M_1−x_O_2_ (M = Ni, Mn, Co, etc.) are superior to layered NCM-like cathode materials due to much greater specific capacities and energies. The high capacity of Li-rich materials is provided by the oxygen redox process in addition to the redox reactions of transition metals (TM) when cycling to a high voltage (above 4.5 V) [4,5,6,7,8,9]. The first charge–discharge cycle of these materials to a high voltage generates a high capacity, however, with a large irreversibility, and leads to a structural transformation, whose nature is the subject of many recent discussions [4,8,10]. In the literature, the structural transformation is attributed to partial losses of oxygen and lithium mainly from the particle surface [3,11,12]. An increase in the discharge capacity is correlated with the formation of an electrochemically active manganese-containing phase [13,14]. During further cycling, TM ions migrate to the lithium sites, which gradually leads to the transformation of the layered structure to the spinel-like one [15,16].

The structure of Li-rich materials is considered in the literature as both a two-phase composite (nanocomposite) consisting of the trigonal (sp. g. $R\bar{3}m)$ and the monoclinic (sp. g. *C*2/*m*) phases [17,18,19,20] and a solid solution based on the monoclinic [21,22] or the trigonal phase [23]. The formation of intergrowth structures and mirror twins is also discussed in the literature [23,24]. The structure complexity, a large first cycle irreversibility, and the structural transformation leading to a voltage decay and capacity fade do not allow one to apply Li-rich cathode materials in practice [10,25]. For the second decade, scientists have actively investigated the mechanisms of degradation of Li-rich cathode material [26,27,28]; however, a number of processes taking place during electrochemical cycling remain unclear.

It should be noted that many controversial studies concerning both the structure and functional properties of the Li-rich materials were published, which is likely due to the influence of many factors such as the synthesis method and conditions, morphology, tap density, etc. [29,30,31]. Moreover, the cell assembly, including the cell components, and the cycling mode, and other factors affect the functional properties of the cathode materials.

To improve the electrochemical properties of the Li-rich cathode material, it is necessary to understand the nature of the structural transformations and to find the methods to suppress unfavorable processes. The variation of the main components (Li, Mn, Ni, Co) is one of the methods to influence on the structure and properties of the Li-rich materials. Herein, we studied an effect of the different Li/(Mn + Ni + Co) ratios from 1.2 to 1.65 on the morphology, structure, and electrochemical properties of the materials such as cathodes for LIBs. We also studied the influence of the different testing modes, including so called formation cycles, in which the upper voltage limit increased gradually from cycle to cycle at different current densities. It was found that the slow formation cycles were required for the better electrochemical performance of the Li-rich oxides. All the materials were also tested in three different voltage ranges to estimate contributions of the low and high voltages to the material degradation. It was found that the cycling to the low voltage limit of 2.5 V was necessary to obtain the high capacity and stable cyclability of the Li-rich cathode materials. 

## 2. Materials and Methods

The cathode materials were synthesized by coprecipitation of the transition metal (nickel, manganese, cobalt) carbonates from the corresponding nitrate salts. Potassium or sodium carbonate was used as a precipitator. The synthesis procedure was described in detail in our previous works [32,33]. The carbonate precursor was thoroughly mixed with lithium hydroxide monohydrate in ethanol and annealed at 480 °C for 6 h and 900 °C for 12 h. The targeted compositions of the cathode materials with sample designations and metal ratios are listed in Table 1.

The cathode materials obtained were characterized by XRD with the structure parameter refinement by the Rietveld method. The X-ray diffraction studies were carried out with a Bruker D8 Advance (Bruker AXS, Germany, Cu Kα, (Ni filter), λ = 0.15418 nm, 40 kW/40 mA, LynxEye 1D detector) diffractometer at room temperature in the 2θ range of 10°–90° with a step of 0.02°. The data collection was performed using the BrukerDIFFRACplus software package (Bruker AXS, Karlsruhe, Germany); the analysis was carried out with the EVA and TOPAS programs. The morphology of the materials was studied by SEM (NVision-40 (Carl Zeiss, Oberkochen, Germany)) with EDX microanalysis. The size particle distribution analysis was performed with the use of a laser particle sizer Analysette 22 MicroTec Plus (Idar-Oberstein, Germany). The compositions of the synthesized compounds were determined by ICP-OES (Thermo Scientific iCAP XP, Waltham, MA, USA).

All the compounds synthesized were tested in CR2032 coin-type cells as the active material (92 wt%). Polyvinylidene difluoride Solef 513 (Solvay, 3 wt%) and carbon black Super C65 (Timcal, 5 wt%) were used as a binder and electroconductive additive, respectively. The lithium foil was used as an anode, and two layers of Celgard 2325 were used as a separator. The used electrolyte (TC-E918, Tinci, Guangzhou, China) contained LiPF_6_ salt dissolved in a mixture of ethylene, diethyl, ethylmethyl and propylene carbonates. 

The electrochemical studies were performed using a Neware CT-4008W-5V10mA battery tester in the galvanostatic cycling mode in the voltage ranges of 2.5–4.7, 2.5–4.3, and 3.0–4.8 V at the current densities of 20 and 80 mA/g. The results of all the electrochemical tests were averaged over 4–6 cells. The formation cycles (by 2 cycles in the ranges of 2.5–4.3, 2.5–4.5, 2.5–4.6, and 2.5–4.7 V, successively) were conducted at the current density of 20 or 80 mA/g before the further cycling tests. The cycling tests at the current density of 80 mA/g without formation cycles were performed in the range of 2.5–4.7 V. The rate capability was examined in the range of 2.5–4.7 V, and the current densities were from 80 to 480 mA/g. 

The galvanostatic intermittent titration (GITT) was performed during discharge process to estimate the resistance values. At first, the formation cycles were also carried out; and then the samples were cycled in the required voltage range. The cell was discharged at a constant current during 30 min succeeded by the current interruption (relaxation time) for 60 min, which is sufficient to achieve an equilibrium voltage value. The steps were repeated four times within a discharge, i.e., full discharge time was 120 min. From the GITT results, we estimated the resistance values by the following procedure. At first, the voltage was measured at the end of each discharge step (*U*1). Then, the cut-off voltage was measured immediately after the current interruption (*U*2) and at the end of relaxation time (*U*3). The cell resistances were calculated from the voltage differences between *U2* and *U*1 (*R_ohm._*), and *U*3 and *U*2 (*R_pol_*_._) according to Equations (1) and (2), respectively:

(1) $$R_{ohm.}=\frac{U2-U1}{I}$$

(2) $$R_{pol.}=\frac{U3-U2}{I}$$

where *I*-discharge current (A).

## 3. Results and Discussion

### 3.1. Chemical and Structural Analyses

The SEM images of the carbonate precursors for the Li-rich materials of different compositions are presented in Figure S1. All precursors were spheric-like agglomerates. The agglomerates became larger in the samples with a larger manganese content. The average agglomerate size of the LMR20 carbonate precursor was 8 µm, but there were also smaller agglomerates (size range of 0.5–1 µm). The average agglomerate size for the LMR35 precursor was about 8 µm, and there were also small agglomerates 1–2 µm in size. The same average value for the LMR50 precursor was 5–12 µm, whereas it was 15–16 µm for LMR65. The narrowest size distribution was observed for the LMR65 carbonate precursor. The average primary particle sizes for the LMR65 and LMR50 precursors were 400–500 and 40–50 nm, respectively. The same values for LMR35 and LMR20 were less than 50 nm.

**Figure 1:**
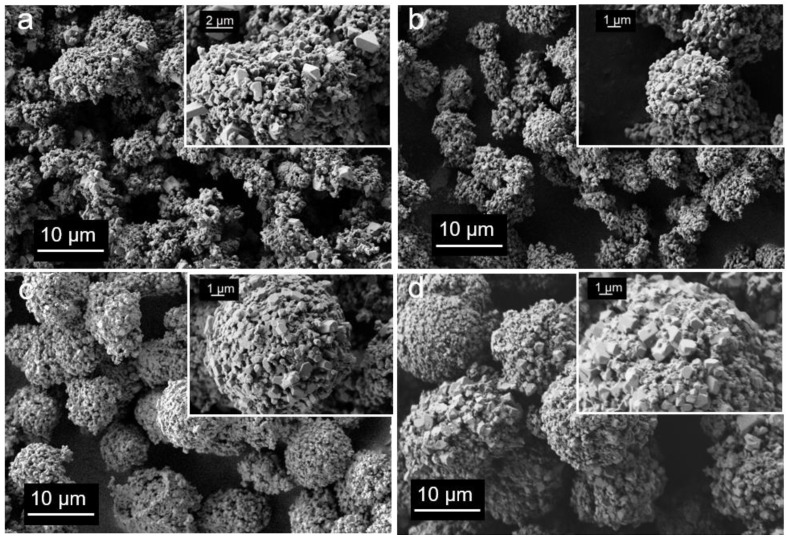
SEM micrographs of the cathode materials at different magnifications: (**a**) LMR20, (**b**) LMR35, (**c**) LMR50, and (**d**) LMR65.

The cathode materials, obtained by a solid-state reaction with lithium hydroxide monohydrate and following annealing, maintained the shape and size of the carbonate agglomerates (Figure 1). The primary particle sizes varied in the ranges of 250 nm–2 µm, 300–800 nm, 400 nm–1 µm, and 400 nm–2.5 µm for LMR20, LMR35, LMR50, and LMR65, respectively. 

The differential size distributions for the cathode materials are shown in Figure S2. The numeric values d10, d50, d90 are listed in Table 2. As is observed, the agglomerates increase with an increase in the manganese content in the material composition. At the same time, the size distributions become narrower. Width of agglomerate size distribution characterized by (d90-d10)/d50 value is very close to LMR50 and LMR65.

The cathode material compositions determined by ICP-OES were close to the targeted compositions (Table S1).

**Figure 2:**
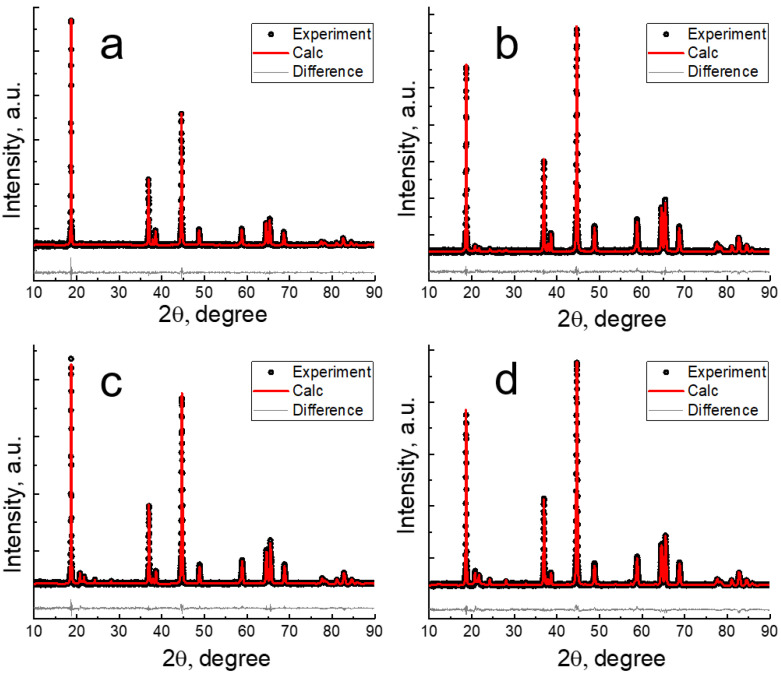
XRD patterns for the cathode materials with different compositions: (**a**) LMR20, (**b**) LMR35, (**c**) LMR50, (**d**) LMR65.

Most of the peaks in the XRD patterns can be described by both the trigonal structure with $R\bar{3}m$ space group and the monoclinic structure with *C*2/*m* space group. In the range of a 20–30° 2θ in the LR35, LR50, LR65 diffractograms, the broadened, low-intensity peaks were observed, characteristic of a superlattice monoclinic phase with ordering of some lithium ions in the transition metal layers (Figure 2). The intensities of these peaks increased in the order of LMR35, LMR50, LMR65. No superstructural peaks were observed in the X-ray pattern of LMR20, which may indicate a small number of the layers with ordered Li ions. We used the model of the solid solution based on the monoclinic phase for the Rietveld refinement (Table 3).

The dependence of the peak broadening on the diffraction angle was described by the Williamson–Hall approach. The larger was the manganese content and the smaller were the nickel and cobalt concentrations in the composition of the cathode materials, the smaller were the unit cell parameters.

### 3.2. Electrochemical Characterization

Three different protocols of the electrochemical tests were used to study the samples. **Protocol 1** included the formation cycles consisting of the successive cycles by two samples in the ranges of 2.5–4.3, 2.5–4.5, 2.5–4.6, and 2.5–4.7 V at the current density of 20 mA/g; **protocol 2** contained the same successive formation cycles at the current density of 80 mA/g; and **protocol 3** constituted the cycling without formation.

**Figure 3:**
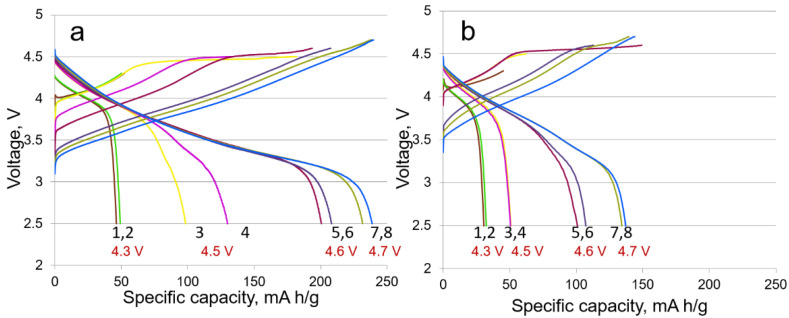
Charge–discharge curves of the formation cycles for LMR50 sample at the current densities of (**a**) 20 mA/g (**protocol 1**) and (**b**) 80 mA/g (**protocol 2**).

A comparison of the formation cycles (LMR50 sample) at different current densities (**protocols 1 and 2**) is presented in Figure 3.

As is seen, the formation cycles at the current density of 80 mA/g have a negative effect on the discharge capacity value compared with the formation cycles at the current density of 20 mA/g. The discharge capacity was about 240 mAh/g at the second formation cycle to 4.7 V (the eighth cycle in total, Figure 3) at the current density of 20 mA/g, whereas the discharge capacity was only 140 mAh/g at 80 mA/g in the same cycle. Notice also that the highest irreversible capacity at 20 mA/g was observed at the first formation cycle at 2.5–4.5 V (the third cycle in total), and the highest irreversible capacity at 80 mA/g (**protocol 2**) was observed in the later cycles (first cycle at 2.5–4.6 V, i.e., the fifth cycle in total). This fact can indicate that the activation of the materials is kinetically hindered.

**Figure 4:**
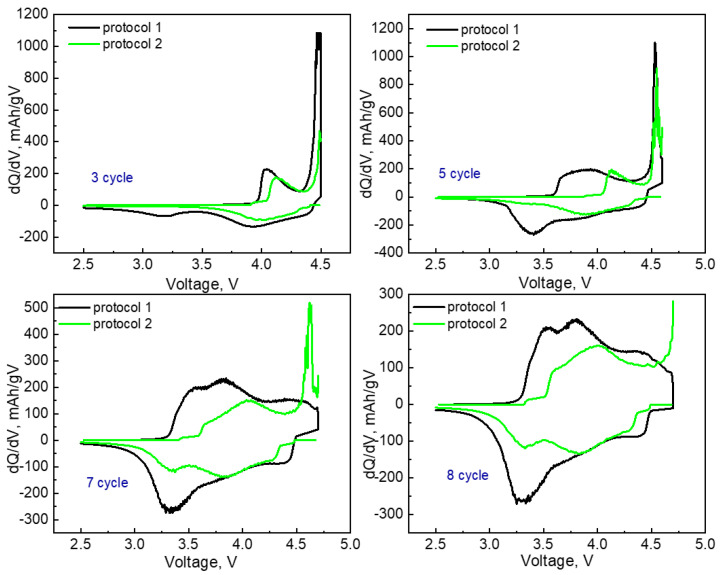
dQ/dV curves of LMR50 sample with different formation protocols.

The differential capacity curves (dQ/dV) for the third, fifth, seventh, and eighth cycles performed according to **protocol 1** and **protocol 2** for the sample LMR50 are shown in Figure 4.

The peak in the anode curve in the region of 4.5–4.6 V is responsible for the oxidation of O^2−^ [34]. This peak appeared later for the samples prepared by **protocol 2**, which correlated with the irreversible capacities due to a partial oxygen loss. The activation also led to the formation of an electrochemically active manganese-containing phase, which manifested itself by additional cathodic and anodic peaks in the range of 3.2–3.4 V [13,35]. These peaks appeared later in case of **protocol 2**. The samples prepared according to this protocol demonstrated worse performance in the course of further cycling. 

All the cathode materials were cycled at the current density of 80 mA/g according to the three different protocols described above. The materials formed by **protocol 2** worked no more than 20 cycles, and their discharge capacities at this current density were about 20–30 mAh/g (the data are not represented graphically).

**Figure 5:**
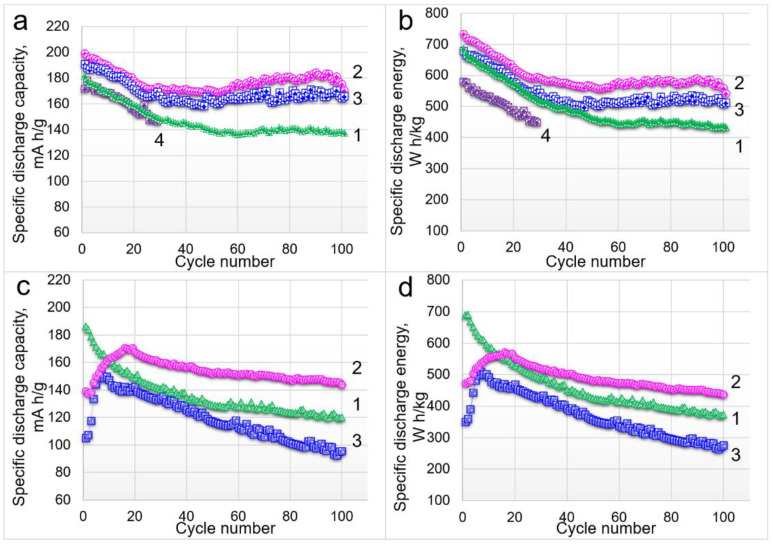
Cycling behavior of the cathode materials with different compositions cycled according to (**a**,**b**) **protocol 1** and (**c**,**d**) **protocol 3** at the current density of 80 mA/g. Designations: 1—LMR20, 2—LMR35, 3—LMR50, and 4—LMR65.

The cycling profiles at 80 mA/g for the oxides tested by **protocol 1** and **protocol 3** are compared in Figure 5. LMR65 had very poor electrochemical performance, so its cycling results are not shown in all the graphs. The specific capacity and energy of LMR50 and LMR35 gradually increased in the first 10–20 cycles (**protocol 3**), whereas for LMR20, these values gradually decreased from the first cycle (Figure 5c,d). The samples after the formation cycles according to **protocol 1** (Figure 5a,b) demonstrated considerably higher specific capacities and energies than the samples without this preliminary procedure. Notice also that LMR35 showed better cyclability compared with the other two samples. In our opinion, the formation cycles with successive voltage increase give a possibility to smooth the inherent transformation of the initial structure in the course of activation. The confirmation of this assumption is the difference in the behavior of LMR50 and LMR35 samples. The structure transformation in LMR35 occurred more gradually (Figure 5c,d), which led to a better cyclability of this material. 

**Figure 6:**
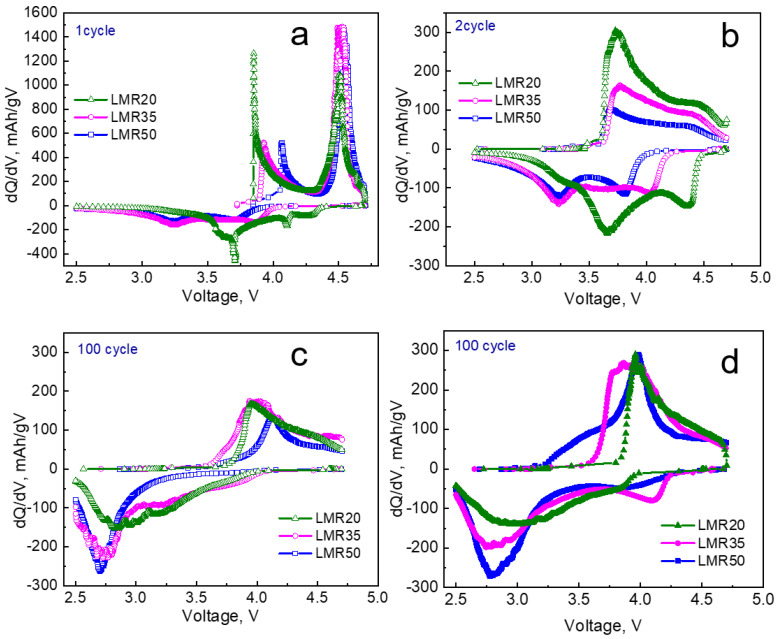
dQ/dV curves for the samples cycled (**a**–**c**) without formation and (**d**) by **protocol 1** at the current density of 80 mA/g.

The dQ/dV curves for the first, second, and 100th cycles for the materials without formation and the 100th cycle for the samples cycled by **protocol 1** are shown in Figure 6.

Apparently, the oxygen oxidation in the samples without formation cycles occurred only in the first cycle (Figure 6a), the anodic peak in the range of 4.5–4.6 V was not observed in the second cycle (Figure 6b). At the same time, the structure transformation continued in the further cycles, as evidenced by increasing the discharge capacity and appearance of the new cathodic peak at 3.2–3.4 V that was described above. This can indicate that an increase in the capacity in the further 10–20 cycles is provided by the manganese-containing species. We did not observe the similar behavior for the sample LMR20 with the lowest manganese concentration from the samples studied. This sample showed only a small shoulder in the area of 3.2–3.4 V to the second cycle and a very broad low-intensity cathodic peak in the area of 2.8–3.2 V to the 100th cycle. 

The dQ/dV curves for the 100th cycle for the same materials without the formation cycles (**protocol 3**) and formed by **protocol 1** are shown in Figure 6c and Figure 6d, respectively. The intensities of the peaks were higher for the samples after preliminary slow formation cycles at 20 mA/g (Figure 6d), which correlated with the higher capacities of these samples. The additional peak in the range of 4.0–4.1 V was observed in the cathodic curve of LMR50 and especially in that of LMR35, where its value was considerably larger (Figure 6d). This peak was attributed in the literature to reversible oxygen activity [36]. Therefore, the LMR35 oxide most likely showed better cyclability and larger values of the discharge capacity (energy) due to better reversibility of the oxygen redox process.

**Figure 7:**
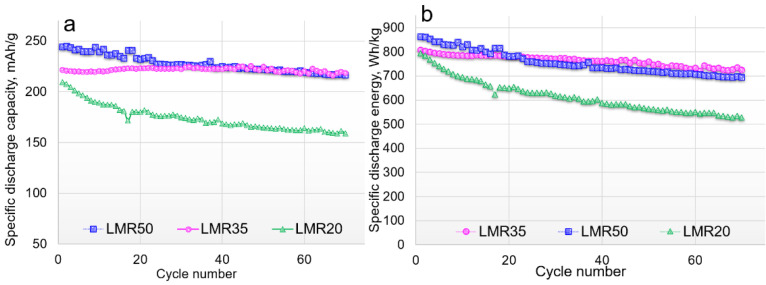
Cycling behavior of cathode materials at 20 mA/g: (**a**) discharge capacity vs. cycle number, (**b**) discharge energy vs. cycle number.

The materials tested by **protocol 1** were also cycled at 20 mA/g (Figure 7).

As was discussed above, LMR65 had very poor electrochemical performance and its data were not shown in the Figure 7. Only 8–10 cycles could be obtained for this material at 20 mA/g. LMR50 showed the highest initial capacity and energy values from the samples under study, but the capacities of LMR35 and LMR50 became comparable to the 20th cycle (Figure 7a,b). At the same time, the energy values for LMR35 material became higher after 20 cycles due to a lower voltage decay. It is significant that the capacity fade for LMR35 material to the 70th cycle was only 1%, which is comparable or better than in the literature data for the Li-rich cathode materials [37,38,39]. The energy fade for LMR35 was 10% to the same cycle because of the voltage decay. The energy fades for LMR50 and LMR20 were 20 and 34%, respectively.

The preliminary formation cycles according to **protocol 1** also positively affected the further cycling at a low rate of 20 mA/g, as was observed for the cycling at the current density of 80 mA/g. However, the formation cycles performed at the high current density (**protocol 2**) worsened the electrochemical performance of the materials compared with the samples cycled both according to **protocol 1** and **protocol 3.** Apparently, the formation cycles at the low current density led to the structure capable to reversibly oxidize/reduce oxygen. 

**Figure 8:**
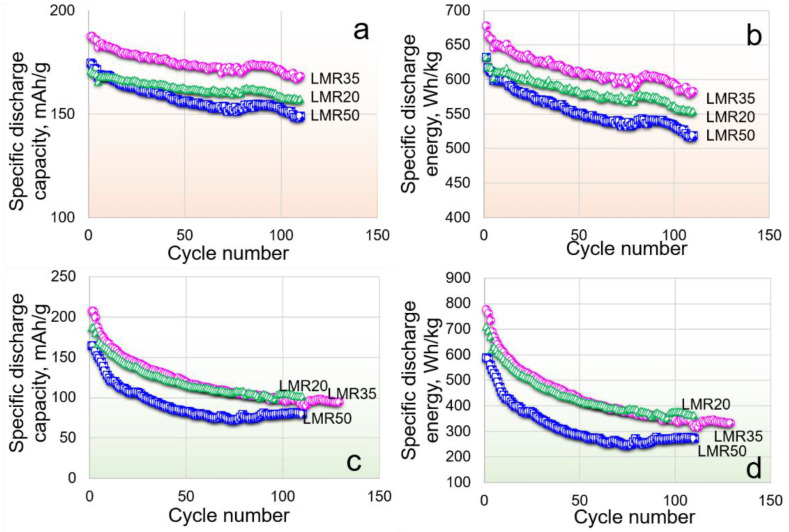
Cycling behavior of the materials in the range of (**a**,**b**) 2.5–4.3 V and (**c**,**d**) 3.0–4.8 V at 80 mA/g.

To study the oxidation/reduction processes taking place during charge/discharge, three compositions (LMR20, LMR35, LMR50) after slow formation cycles (**protocol 1**) were cycled in the three different voltage ranges of 2.5–4.3, 3.0–4.8 (Figure 8), and 2.5–4.7 V (Figure 5a,b). Note that a comparison of the cycling behavior in the voltage ranges of 2.5–4.7 and 2.5–4.8 V showed the minimal difference in the capacity values and capacity retentions for all the samples. The range of 2.5–4.3 V reflects the effect of an increase in the resistance due to a deep discharge. The range of 3.0–4.8 V shows the effect of deep charges, which may contribute to the structural transformations. During cycling in the wide voltage range of 2.5–4.7 V both factors may contribute to the capacity fade.

The comparison of the cycling profiles in the different voltage ranges showed that the capacity fade was not maximal in the widest voltage range of 2.5–4.7 V, as might be expected. However, we observed the maximum of the capacity fade in the 3.0–4.8 V voltage range. The capacity retention for all the materials to the 110th cycle was only about 50% for the 3.0–4.8 V voltage range (Figure 8c,d). The capacity retentions to the same cycle in the ranges of 2.5–4.7 and 2.5–4.3 V were, respectively, 76, 90, and 87% and 92, 89, and 85% for LMR20, LMR35, and LMR50. It should be noted, that the capacity retentions for LMR35 and LMR50 were somewhat lower in the range of 2.5–4.3 V than those in the voltage range of 2.5–4.7 V. The sample LMR20, on the contrary, showed the better cyclability in the range of 2.5–4.3 V. This correlates with the lowest manganese content in this sample, which formally corresponds to the lowest content of the monoclinic phase (or the ordered layers containing transition metals and Li ions). The voltage decay is maximal for all the cathode materials in the 2.5–4.7 V voltage range due to formation of the electrochemically active phase with a lower redox potential and an increase in the polarization resistance in the course of cycling. 

**Figure 9:**
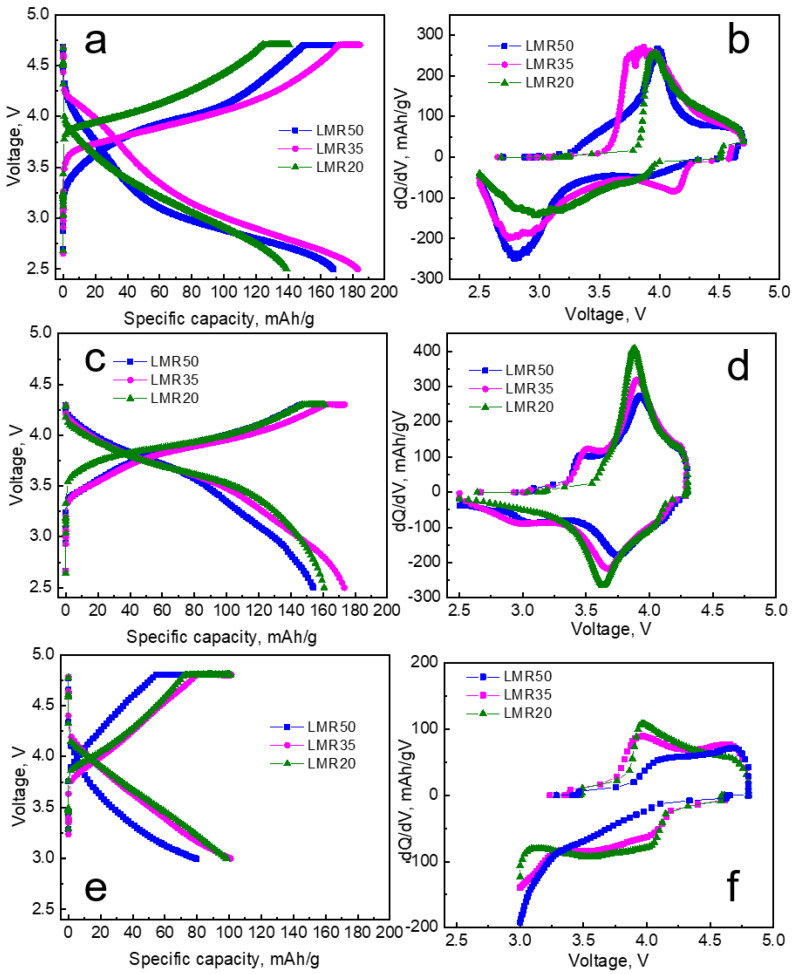
(**a**,**c**,**e**) Charge–-discharge profiles and (**b**,**d**,**e**) dQ/dV curves in the 100th cycle for the different compositions in the voltage ranges of 2.5–4.7, 2.5–4.3, and 3.0–4.8 V, respectively; the current density is equal to 80 mA/g.

The charge–discharge profiles and the dQ/dV curves in the 100th cycle for the three different voltage ranges are shown in Figure 9.

The redox peaks in the dQ/dV curves (Figure 9b,d) for all the samples cycled in the ranges of 2.5–4.3 and 2.5–4.7 V were observed at different potentials. The cathodic peak for the samples cycled in the range of 2.5–4.7 V was broadened and shifted to lower potentials, whereas that for the 2.5–4.3 V range slightly changed with cycling. The LMR35 sample had the additional cathodic peak at 4.1–4.2 V in the range of 2.5–4.7 V. The discharge capacities obtained in the range of 3.0–4.8 V in the 100th cycle for all the samples were considerably lower (Figure 9e) than those in the voltage ranges of 2.5–4.3 and 2.5–4.7 V. The redox process corresponding to the low-voltage species could not occur in this range, and the related cathodic peaks were not displayed in the dQ/dV plots (Figure 9f). Therefore, to carry out the cycling tests at the lower potential limit higher than 2.5 V is impractical. 

The polarization resistances were calculated for all the cathode materials in the different voltage ranges from the GITT data. As a rule, the polarization resistance characterizes the processes associated with the surface concentration changes due to the hindered diffusion of lithium ions from the particle surface to the bulk [40]. 

**Figure 10:**
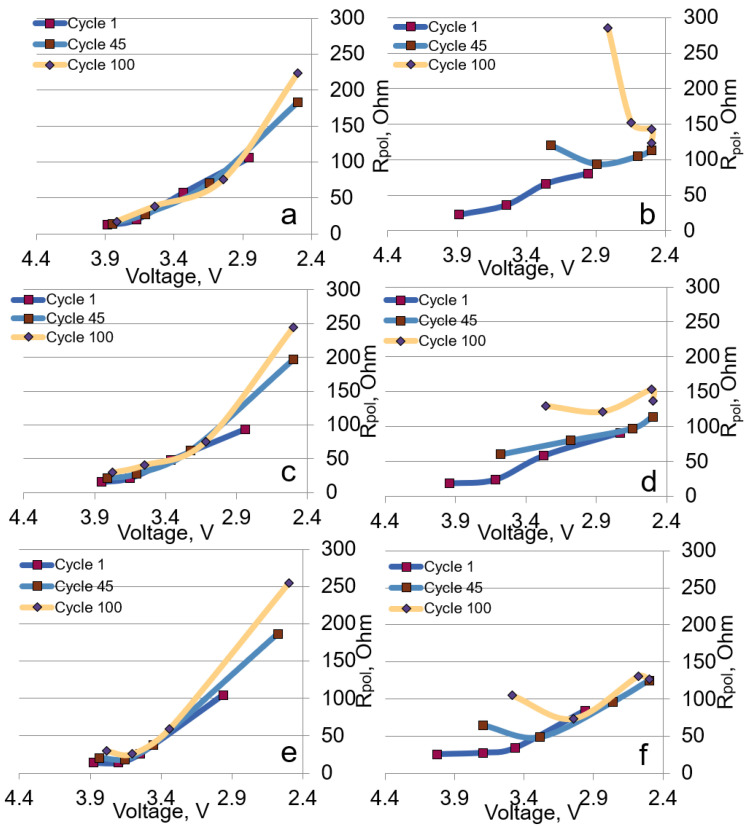
Polarization resistance values on discharge during cycling in the ranges of 2.5–4.3 V ((**a**) LMR50, (**c**) LMR35, (**e**) LMR20) and 2.5–4.7 V ((**b**) LMR50, (**d**) LMR35, (**f**) LMR20).

The polarization resistances calculated for the cell discharge vs. the voltage values are shown in Figure 10. In the voltage range of 2.5–4.3 V, the resistance increased in the course of discharge in each cycle for all the samples. In the range of 2.5–4.7 V, the character of the curves was more complex and varied depending on the cycle number for all the materials. In this range, we also observed an increase in the resistance during the first cycle. However, from the 45th cycle, the character of the resistance dependence changed within a cycle showing an increase at both the beginning and the end of the cycle. At the same time, the resistance values were lower in the wide voltage range than those in the range of 2.5–4.3 V. The least changes within a cycle were observed for LMR35, which demonstrated the better cyclability.

The rate capabilities in the range of 2.5–4.7 V were studied for all the materials with the different compositions (Figure S3). The LMR35 sample showed a significantly lower discharge capacity fade at the high current densities compared to the other samples. 

## 4. Conclusions

The effect of the different Li-rich compositions on the properties and electrochemical performance of the materials was investigated; the Li/(Mn + Ni + Co) ratios were varied from 1.2 to 1.65. The oxides were synthesized by coprecipitation of TM carbonates followed by a solid-state reaction with a lithium source. Different schemes of the electrochemical tests were used to study these materials. Some of the tests included so-called formation cycles—eight cycles with gradual increasing of the upper voltage limit at the different current densities. In addition, we studied also the electrochemical behavior of the materials in the three voltage ranges, namely, 2.5–4.3, 2.5–4.7, and 3.0–4.8 V. It was found that successive formation cycles at the low current density (20 mA/g, 0.1C) are necessary to obtain the high discharge capacities and stable cycling of the materials under study. The electrochemical tests in the three different voltage ranges were performed after preliminary formation cycles described above. The results of this study showed that the largest capacity fade and the lowest capacities during cycling are observed in the range of 3.0–4.8 V, whereas the cycling in the widest voltage range of 2.5–4.7 V demonstrates very good cyclability with the high discharge capacities. This behavior of the materials under study may be explained by the formation of the electrochemically active phase with the redox processes at a low potential as a result of the structural transformations during the first cycles. These structural changes, although leading to a voltage decay, provide an additional discharge capacity. In addition, the structures formed by the slow preliminary formation revealed a greater reversibility of the anionic redox process, which also might contribute to the discharge capacity. Comparing the 2.5–4.3 V and 2.5–4.7 V ranges, it may be noted that the polarization resistance in the 2.5–4.3 V range increased significantly within one discharge cycle, as opposite to that when the samples cycled in the 2.5–4.7 V range. In this voltage range, the resistance values were lower (except LMR50) than those the 2.5–4.3 V range. The LMR35 sample (Li/(Mn + Ni + Co) = 1.35) showed the best electrochemical properties among all the other samples, likely due to a lower polarization resistance, a more gradual structure transformation, and a greater reversibility of the oxygen redox process. 

## Figures and Tables

**Table 1:** Designations of the samples and their targeted compositions.

Samples	Targeted Composition	Li/(Mn + Ni + Co)
LMR20	Li_1.091_Ni_0.242_Mn_0.424_Co_0.242_O_2_	1.20
LMR35	Li_1.149_Ni_0.184_Mn_0.482_Co_0.184_O_2_	1.35
LMR50	Li_1.200_Ni_0.134_Mn_0.534_Co_0.134_O_2_	1.50
LMR65	Li_1.245_Ni_0.088_Mn_0.579_Co_0.088_O_2_	1.65

**Table 2:** The agglomerate size distribution for the Li-rich samples.

	LMR20	LMR35	LMR50	LMR65
d10, μm	3.50	4.56	6.23	8.75
d50, μm	8.11	8.54	10.88	16.97
d90, μm	15.97	15.26	17.64	27.15
(d90-d10)/d50	1.54	1.25	1.05	1.08

**Table 3:** Crystallographic data and the unit cell parameters from the Rietveld refinement (*C*2/*m* space group) for the cathode materials.

	LMR20	LMR35	LMR50	LMR65
Rwp, %	1.76	2.84	2.10	2.40
Rp, %	1.28	2.22	1.60	1.81
GOOF, %	3.19	5.05	4.58	5.59
a, Å	4.9441(4)	4.9363(4)	4.9317(4)	4.9305(3)
b, Å	8.5637(8)	8.5502(6)	8.5422(8)	8.5401(6)
c, Å	5.0245(14)	5.0218(4)	5.0216(4)	5.0233(4)
β, Å	109.226(3)	109.264(3)	109.283(2)	109.303(2)
V, Å^3^	200.87(7)	200.08(3)	199.68(4)	199.63(3)

## Supplementary Materials

The following supporting information can be downloaded at: https://www.mdpi.com/article/10.3390/nano12224054/s1, Figure S1: Micrographs of carbonate precursors for cathode materials with different magnifications: (a,b) LMR20, (c,d) LMR35, (e,f) LMR50, (g,h) LMR65; Figure S2: Differential agglomerate distribution of cathode materials with different compositions: (a) LMR20, (b) LMR35, (c) LMR50, (d) LMR65; Figure S3: Rate capabilities of the cathode materials; Table S1: Determined by ICP-OES compositions of cathode materials.
