# Supplementary material for: Influence of the Composition and Testing Modes on the Electrochemical Performance of Li-Rich Cathode Materials
Source: Nanomaterials (Basel). 2022 Nov 17;12(22):4054. doi: 10.3390/nano12224054 (PMC9693166; doi:10.3390/nano12224054)
Supplement: Supplementary file 1 [file nanomaterials-12-04054-s001.zip › nanomaterials-2011933-supplementary.pdf]

Supplementary

Influence of the Composition and Testing Modes on the Electrochemical Performance of Li-Rich Cathode Materials

Lidia Pechen [1,*], Elena Makhonina [1], Anna Medvedeva [1], Yury Politov [1], Aleksander Rumyantsev [2], Yury Koshtyal [2], Alexander Goloveshkin [3], and Igor Eremenko [1,*]

1 Kurnakov Institute of General and Inorganic Chemistry of Russian Academy of Sciences, 31 Leninsky pr., 119991 Moscow, Russia
2 Ioffe Institute of Russian Academy of Sciences, 26 Politekhnicheskaya ul., 194021 St Petersburg, Russia
3 A.N. Nesmeyanov Institute of Organoelement Compounds of Russian Academy of Sciences, 28 Vavilova ul., 119334 Moscow, Russia
* Correspondence: lidia.s.maslennikova@gmail.com; Tel.: +74957756585 (ext. 2-41)

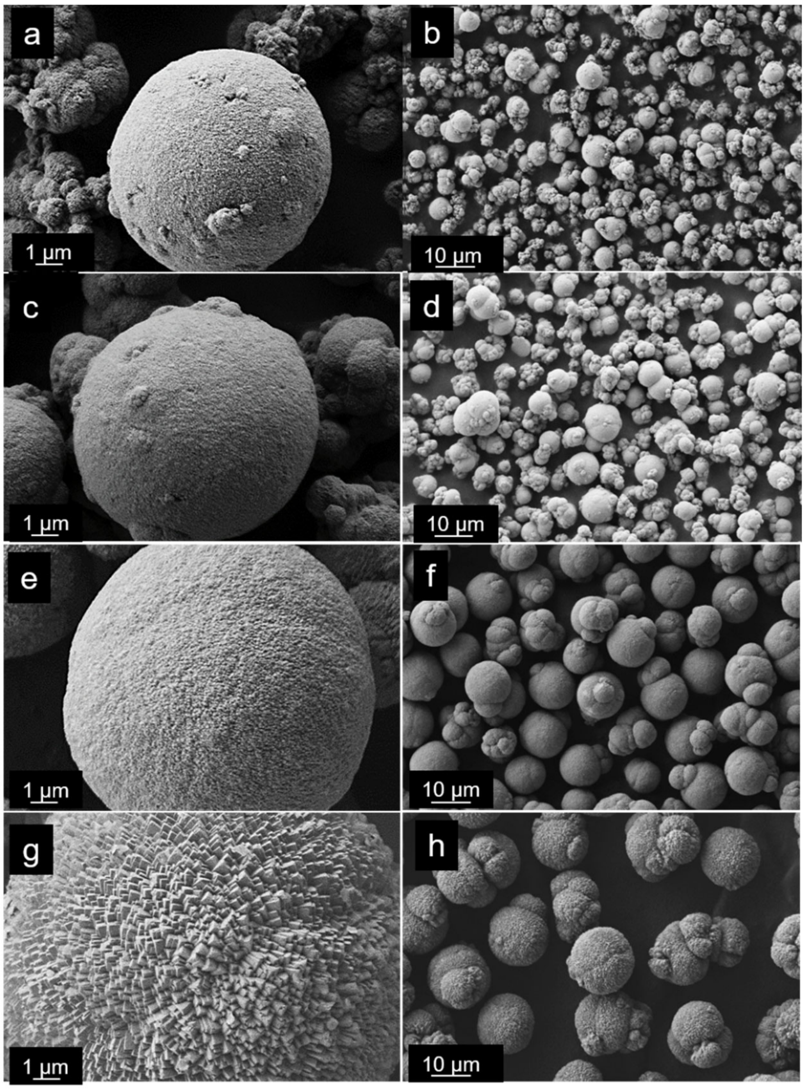

Figure S1. SEM images of the carbonate precursors for cathode materials with different magnifications: (a, b) LMR20, (c, d) LMR35, (e, f) LMR50, and (g, h) LMR65.

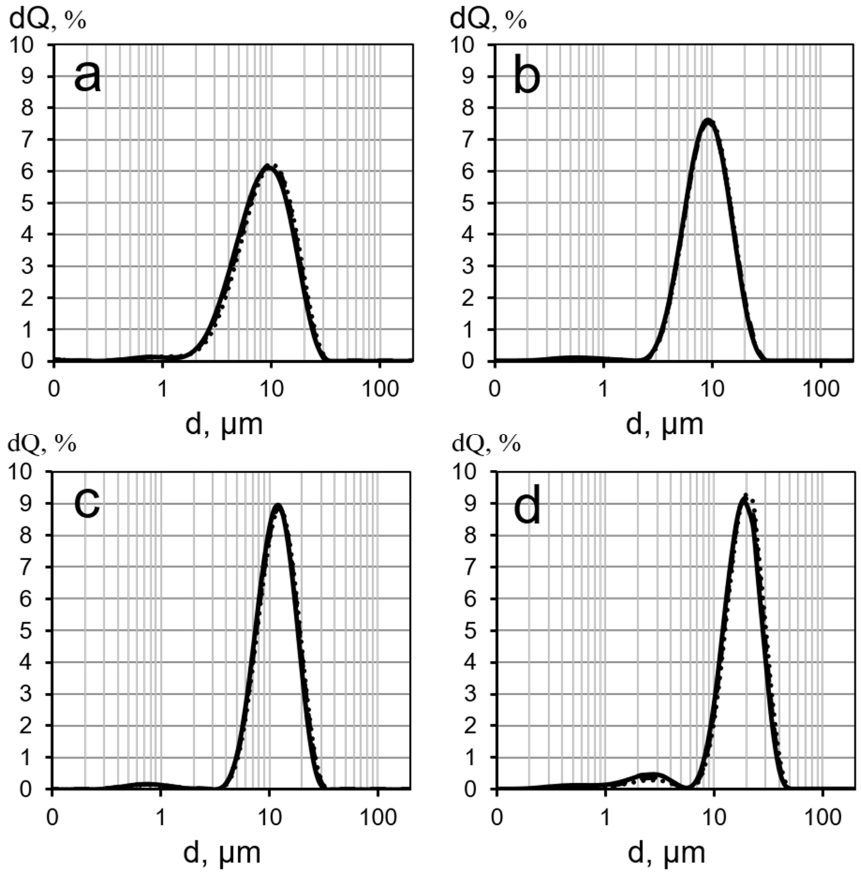

Figure S2. Differential agglomerate distributions for cathode materials with different compositions: (a) LMR20, (b) LMR35, (c) LMR50, and (d) LMR65.

Table S1. Cathode material compositions determined by ICP-OES

Samples	Determined composition
LMR20	$Li_{1.109}Ni_{0.244}Mn_{0.424}Co_{0.242}O_2$
LMR35	$Li_{1.147}Ni_{0.184}Mn_{0.483}Co_{0.185}O_2$
LMR50	$Li_{1.210}Ni_{0.133}Mn_{0.535}Co_{0.135}O_2$
LMR65	$Li_{1.249}Ni_{0.088}Mn_{0.579}Co_{0.087}O_2$

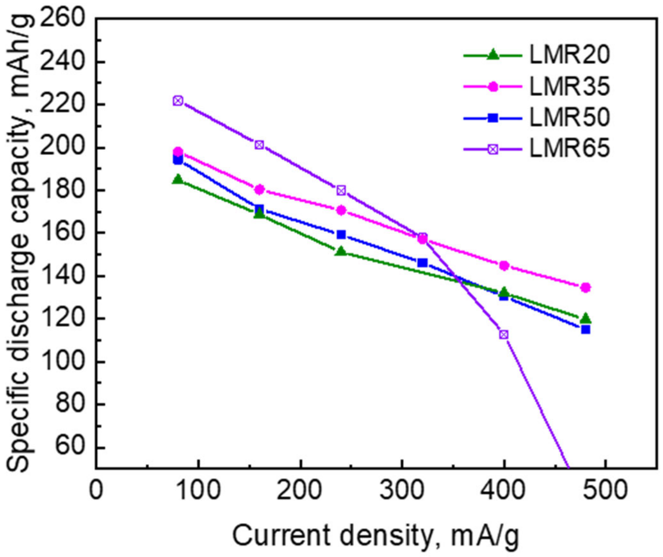

Figure S3. Rate capabilities of the cathode materials.
